# Involvement of Renal Corpuscle microRNA Expression on Epithelial-to-Mesenchymal Transition in Maternal Low Protein Diet in Adult Programmed Rats

**DOI:** 10.1371/journal.pone.0071310

**Published:** 2013-08-19

**Authors:** Letícia de Barros Sene, Flávia Fernandes Mesquita, Leonardo Nazário de Moraes, Daniela Carvalho Santos, Robson Carvalho, José Antônio Rocha Gontijo, Patrícia Aline Boer

**Affiliations:** 1 Fetal Programming Laboratory, Morphology Department, São Paulo State University, Botucatu, São Paulo, Brazil; 2 Striated Muscle Laboratory, Morphology Department, São Paulo State University, Botucatu, São Paulo, Brazil; 3 Renal Function Laboratory, Internal Medicine Department, State University of Campinas, Campinas, São Paulo, Brazil; 4 Morphology Department, São Paulo State University, Botucatu, São Paulo, Brazil; University of Houston, United States of America

## Abstract

Prior study shows that maternal protein-restricted (LP) 16-wk-old offspring have pronounced reduction of nephron number and arterial hypertension associated with unchanged glomerular filtration rate, besides enhanced glomerular area, which may be related to glomerular hyperfiltration/overflow and which accounts for the glomerular filtration barrier breakdown and early glomerulosclerosis. In the current study, LP rats showed heavy proteinuria associated with podocyte simplification and foot process effacement. TGF-β1 glomerular expression was significantly enhanced in LP. Isolated LP glomeruli show a reduced level of miR-200a, miR-141, miR-429 and ZEB2 mRNA and upregulated collagen 1α1/2 mRNA expression. By western blot analyzes of whole kidney tissue, we found significant reduction of both podocin and nephrin and enhanced expression of mesenchymal protein markers such as desmin, collagen type I and fibronectin. From our present knowledge, these are the first data showing renal miRNA modulation in the protein restriction model of fetal programming. The fetal-programmed adult offspring showed pronounced structural glomerular disorders with an accentuated and advanced stage of fibrosis, which led us to state that the glomerular miR-200 family would be downregulated by TGF-β1 action inducing ZEB 2 expression that may subsequently cause glomeruli epithelial-to-mesenchymal transition.

## Introduction

The Barker theory has proposed that any adverse event during intrauterine development induces response in the fetus, which has altered its phenotype predisposition to cardiovascular disease in later life [1]. Thus, environmental as well as genetic factors can interfere on organ development leading to dysfunctional and/or structural effects in tissues and organs. Nutritional restriction may result in intrauterine growth retardation (IUGR) associated with impaired nephrogenesis and nephron underdosing [2], [3].

Recently, we found that offspring from mothers submitted to gestational low protein diet, at 16-wk-old, showed pronounced reduction of nephron number (27%) associated with decreased fractional urinary sodium excretion and hypertension when compared with the control-diet age-matched group [2], [3]. These results occurred despite unchanged glomerular filtration rate and 17% enhanced glomerular tuft area, thus suggesting that prior tubular dysfunction with enhanced water and sodium reabsorption might, at least in part, be responsible for programming of adult hypertension. However, these morphological and functional changes could be also attributed to a reduced nephron number associated with glomerular hyperfiltration/overflow that may account for the glomerular filtration barrier breakdown and early glomerulosclerosis [2], [3] in low protein diet offspring.

Irreversible renal fibrosis is a common consequence after most renal injuries [4], [5]. Extracellular matrix (ECM) protein deposition in renal tissue is regulated by Transforming Growth Factor-β (TGF-β) [6]. Increased expressions of TGF-β mRNA in podocytes and ECM protein deposition in glomeruli have been found in focal segmental glomerulosclerosis (FSGS) [7], membranous nephropathy [8], [9] and diabetic nephropathy [10]. Also, TGF-β1 enhances the expression of ZEB1/2, which is the repressor of genes such as E-cadherin and collagens [5], [11], [12].

Type II epithelial-to-mesenchymal transition (EMT) is associated with fibrosis progression [13], and a number of studies implicate altered expression of several miRNAs with the phenotypic changes that occurs during EMT, in the development of fibrosis [14]–[17] and in progressive kidney disease [15]. The miRNAs, short (∼22 nucleotides) noncoding RNAs, induce post-transcriptional gene repression by blocking protein translation and binding themselves to the 3′ untranslated region (UTR) of their target genes or by mRNA degradation. Therefore, they have a potential role on gene expression under physiological and pathological conditions. Members of the miR-200 family and miR-192 act as protectors of the normal epithelial phenotype and are markedly downregulated in TGF-β-induced EMT [16], [17]–[21].

Despite several changes observed in the kidney structure and function of adult maternal LP offspring, the renal pattern of miRNA expression in this model remains unknown. Since the renal tissue is composed of different mesenchymal and epithelial cell types, and the EMT process may be uneven throughout the whole renal parenchyma, the study of isolated renal corpuscle could be much more specific and reliable. The renal corpuscles are constituted by four resident cell types: mesangial, endothelial, visceral (podocytes) and parietal epithelial cells (PECs) that have particular glomerular functions and express specific proteins. Podocytes are terminally differentiated epithelial cells that have a very low proliferative capacity [22] and, thus, injury and/or loss of these cells can lead to proteinuria and glomerular scarring [4]. Podocytes cannot proliferate and regenerate, but recently Appel *et al.* have postulated that PECs may migrate to the glomerular tuft and differentiate into podocyte, but how this event occurs is unknown [23].

In this study, the transmission and scanning electron microscopy (TEM/SEM) were used to characterize the morphological disorders in renal corpuscles in adult 16-wk-old male offspring from maternal low protein intake compared with age-matched controls. We also verified the expression of miRNAs and mRNAs in isolated renal corpuscles prepared from programming adult offspring compared with appropriated controls. Protein markers of fibrosis and EMT and protein urinary excretion rates were also evaluated.

## Results

### Rats and Kidney Mass Weight

**Figure 1:**
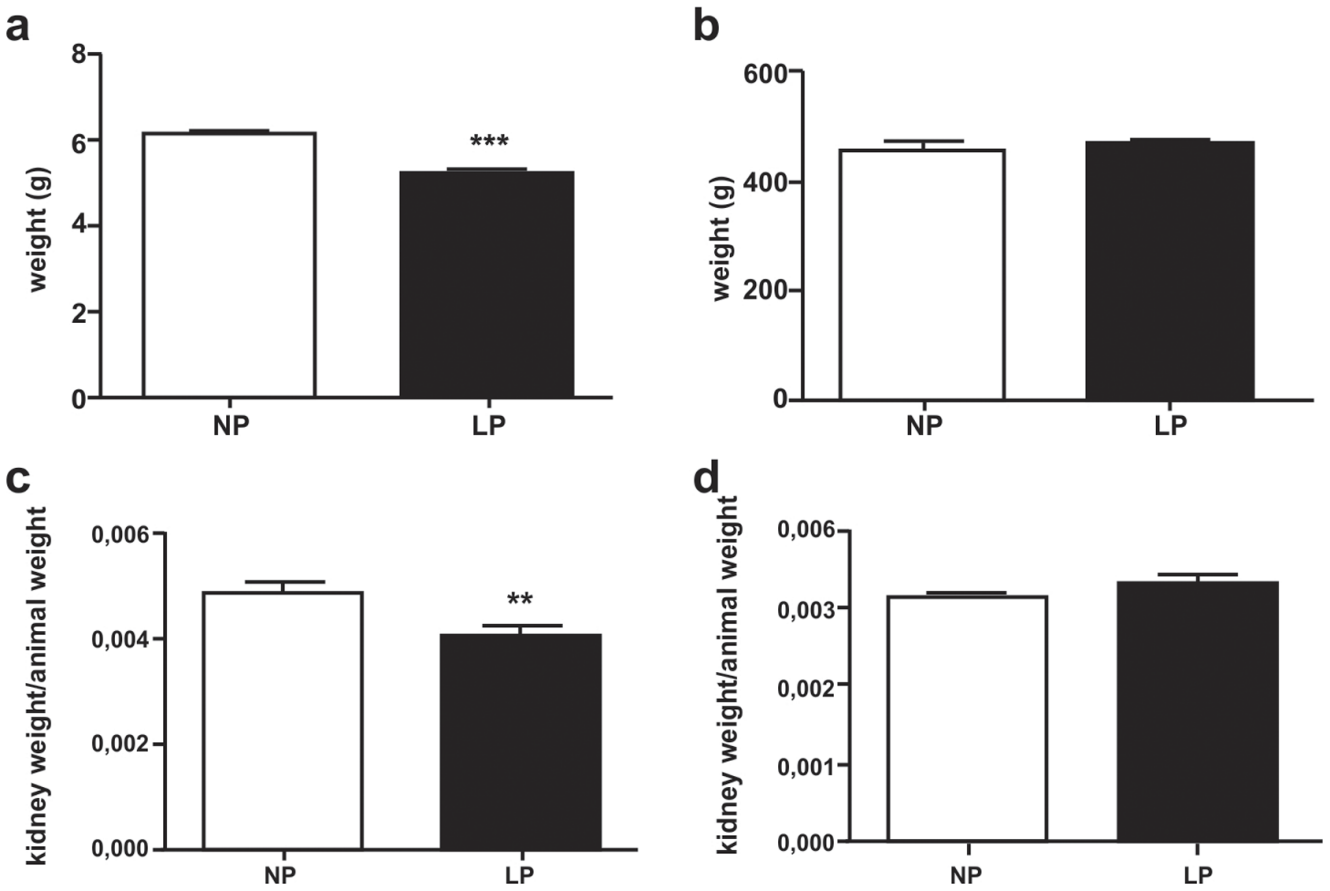
Male 16-wk LP (n = 10) offspring body and kidney weights compared to age-matched control (n = 10). (**a**) Birth weight; (**b**) Adult (16-wk-old) offspring; (**c**) Right kidney weight in 16-wk-old LP compared to NP rats; and, (**d**) Left kidney weight in 16-wk-old animals.**p = 0.005, ***p<0.0001.

Birth weight in male offspring was significantly lower in LP (n = 10, Figure 1a) compared to control group (n = 10). However, at 16-wk of life, the animals showed no difference in weight when compared NP (n = 10) and LP (n = 10) offspring (Figure 1b). Right kidney weight was significantly lower in LP group (n = 10, Figure 1c) compared to control (n = 10); however, left kidney weight was similar in both groups (Figure 1d).

### Effect of Maternal Low Protein Diet on Offspring Proteinuria and Serum Creatinine Levels

**Figure 2:**
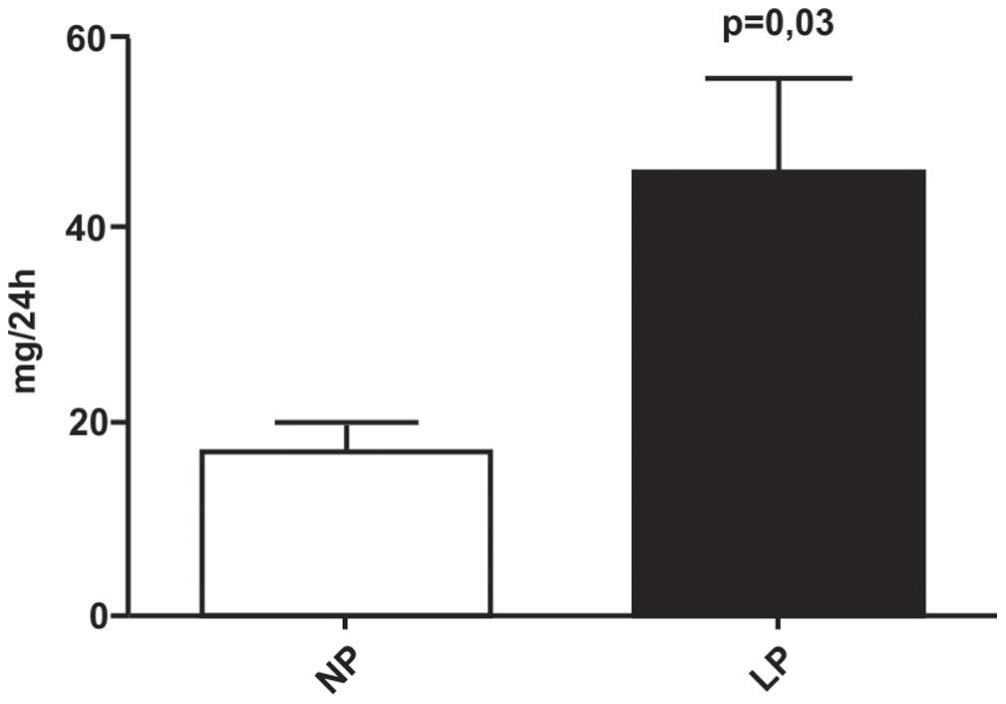
Daily urinary protein excretion in 16-wk-old LP offspring (n = 10) compared to appropriated age-matched controls (n = 10).

Urine from LP rats (45.92±19.6 mg/day, n = 10) showed an elevated protein level when compared with age-matched NP rats(17.41±4.9 mg/day, n = 10), as shown in Figure 2. For these same experimental groups, no changes were observed for creatinine serum levels (0.47±0.02 *vs*. 0.50±0.02 mg/dL).

### Effect of Maternal Low Protein Diet on Glomeruli Ultrastructure

**Figure 3:**
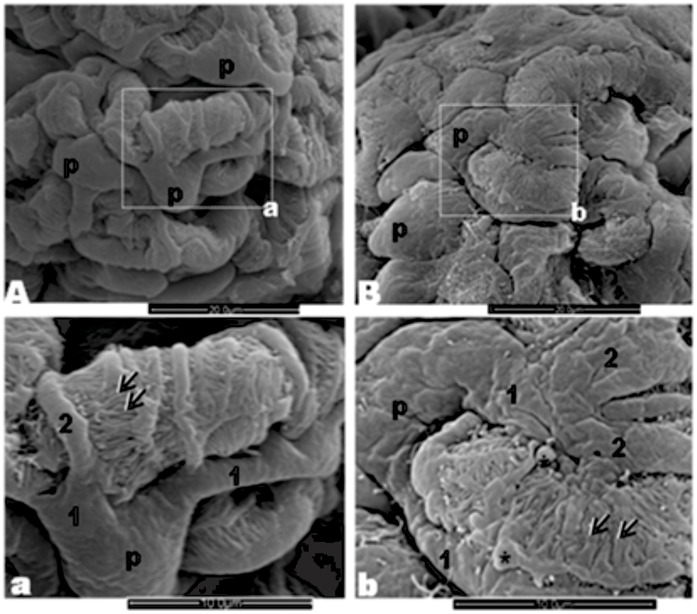
Effect of maternal low protein diet on offspring glomeruli (from 5 LP rats) ultrastructure A: Three-dimensional organization of the outer surface of podocytes (p) surrounding capillaries in a control rat (from 5 NP rats). **a**: Detail of a podocyte showing primary (1) and secondary (2) processes and pedicels (arrow) among which filtration slits can be seen. **B**: LP rat glomerulus with an intensive cohesive arrangement and bulbous and crushed podocytes **b**: Note the irregular surface of cell body with enlarged processes, pseudo cists formation (*), width and club-shaped pedicels and reduced number of filtration slits.

**Figure 4:**
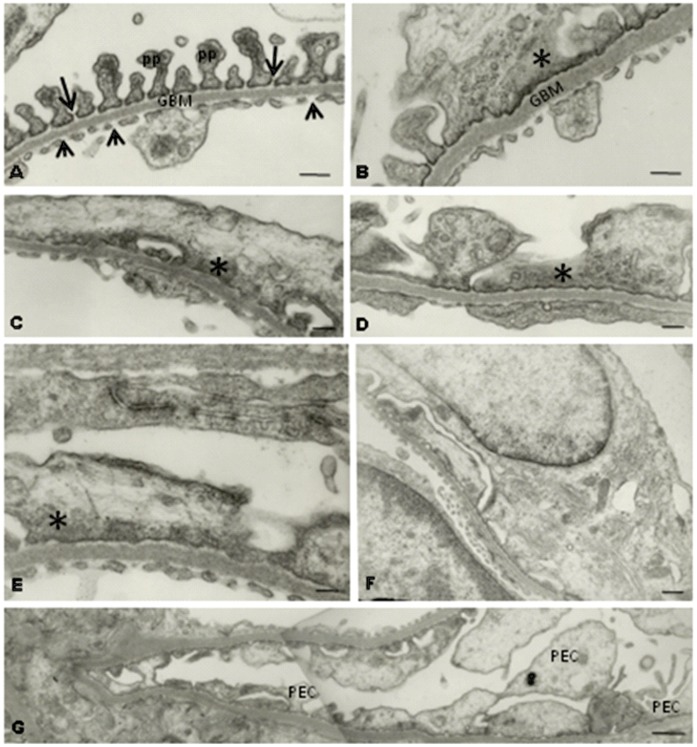
Transmission electron micrographs showing: A) the filtration barrier from NP glomeruli (from 5 rats) showing all the structural components of the filtration barrier, including the fenestrations of the glomerular endothelium (arrowheads), the GBM, the podocyte pedicels (pp) and the filtration slits crossed by tenuous diaphragm membranes (arrows). B–F) the filtration barrier from LP glomeruli (from 5 rats) showing a drastic decrease in the number of pedicels and slit diaphragms. Irregular electron-dense masses occur in the podocyte cytosol. Note in B the increased thickness of GBM compared to what is observed in A–G) in LP glomeruli, we found PECs in the process of differentiating into podocytes.

The normal glomerular visceral epithelium ultrastructure from NP offspring (n = 5) can be seen, by SEM, in Figures 3A and a. This elaborate epithelium was anchored to the outer surface of the thick underlying glomerular basement membrane (GBM) surrounding the glomerular endothelium. In LP offspring (n = 5) glomeruli, we observed an intensive cohesive arrangement with bulbous and crushed podocytes and effacement of foot processes, indicating reduced number of filtration slits (Figures 3B and b). TEM showed that in NP rats the filtration barrier maintains the interdigitating pedicels bridged by the filtration slit membrane, the GBM and the thin fenestrated endothelium (Figure 4A). However, this regular arrangement of the podocyte foot processes completely disappears in LP. The podocytes showed intense simplification, with loss of the filtration slits and the appearance of wide, irregular profiles at the terminal ends of the pedicels (Figure 4B–F). There was a reduction in the complexity of cell–cell connections as a result of podocyte foot process effacement, which ranged from partial retraction of the foot processes to total disappearance of the usual interdigitated pattern. The GBM shows an increased thickness in glomeruli of LP (Figure 4B) when compared to age-matched NP (Figure 4A). At the glomerular parietal epithelial cells (PECs), we found transitional cells at the glomerular vascular stalk that exhibit features of both PECs and podocytes (Figure 4G).

### Immunohistochemistry and Western Blot Analysis

**Figure 5:**
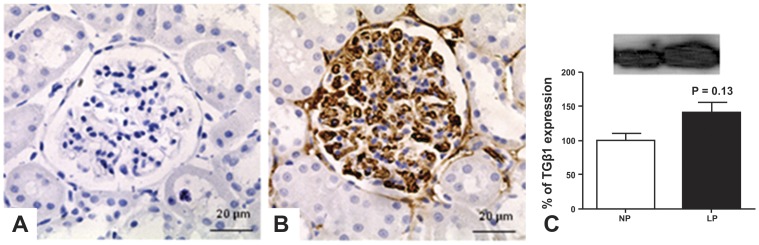
We found an intensive rise of the TGF-β1 expression in LP (B, from 5 rats) compared to NP glomeruli (A, from 5 rats). By western blot from total renal tissue extract we also found enhancement in the expression of this protein in LP offspring (C).

We observed an intensive rise of the TGF-β1 expression in LP glomeruli located in the basement membrane under parietal epithelium (Figure 5B) compared to NP glomeruli expression (Figure 5A). In LP offspring, the TGF-β1 is also roughly expressed in the glomerular basement membrane delimiting glomerular capillaries (Figure 5B). By western blot we also observed enhanced TGF-β1 expression when compared with NP rats at same age (Figure 5C).

**Figure 6:**
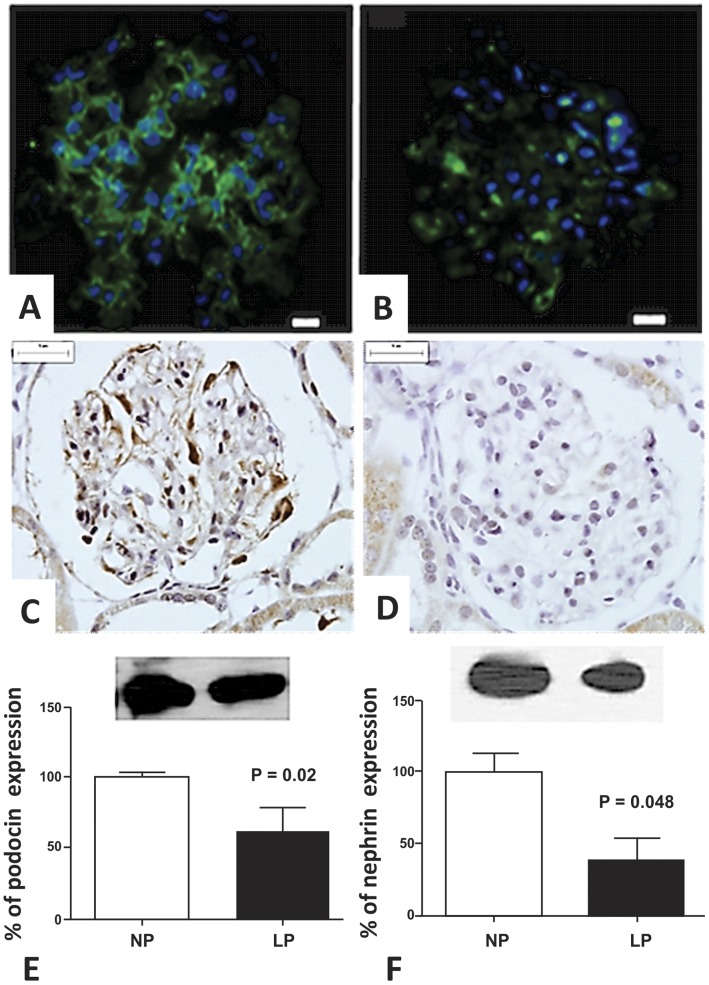
Immunohistochemistry and western blot showing reduction in the expression of glomerular epithelial markers. We can observe an extensive immunoreactivity for nephrin along NP glomerulus (from 5 rats) (A) compared to weak immunostained glomerulus in LP (from 5 rats) (B). E-cadherin glomerular expression was also reduced in LP (D) compared with NP(C) glomeruli. By western Blot from total renal tissue extract we found reduced expression of podocin (E, 39%) and nephrin (F, 62%) in LP.

**Figure 7:**
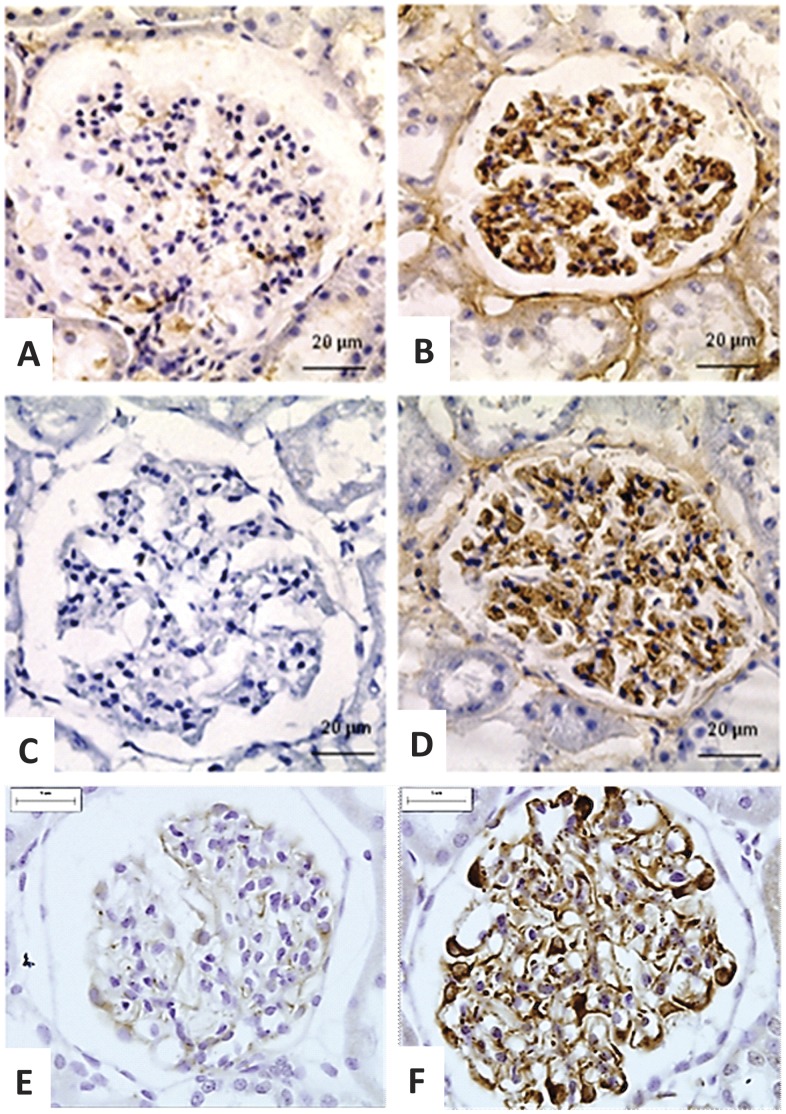
Immunohistochemistry showing enhanced glomerular expression of mesenchymal markers. We verified that fibronectin (B), type I collagen (D) and desmin (F) immunoreactivity are intensively enhanced in LP (n = 5) when compared to that observed in NP (n = 5) (A, C and E, respectively).

**Figure 8:**
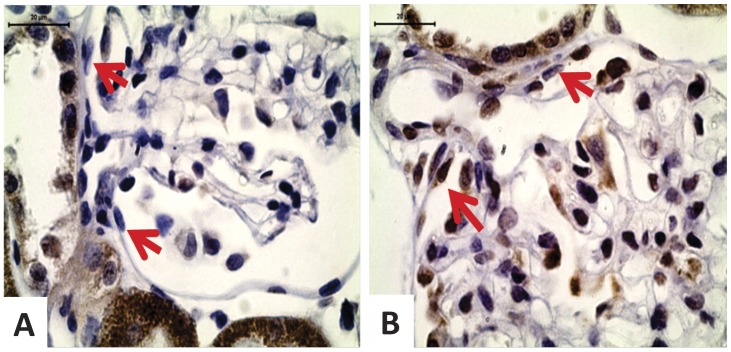
Immunohistochemistry showing enhanced glomerular expression of ZEB 2, an EMT-related mediator. In NP (n = 5), we verified little immunoreactivity for ZEB2 in podocytes and none in PECs (A). In LP (n = 5), both podocytes and PECs (arrows) in the vascular stalk present intensive reactivity (B).

We can observe an extensive immunoreactivity for nephrin along NP glomerulus compared to weak immunostained glomerulus in LP offspring (Figures 6 A and B). We also found a reduction of glomerular E-cadherin immunoreactivity in LP glomerulus (Figures 6C and D). By western blot, we verified that the kidney of 16-wk-old LP rats shows a significant reduction of both podocin and nephrin (Figures 6E and F). The study shows that fibronectin immunoreactivity is accentuated in LP glomeruli (Figures 7A and B) as well as type I collagen expression, highly expressed in LP and conversely to NP offspring glomeruli (Figures 7C and D). Desmin is a mesenchymal marker in podocyte lesion and we found enhanced glomerular desmin immunostaining in LP when compared to what was observed in NP (Figures 7E and F). Additionally, we found enhanced ZEB 2 expression in podocytes and PECs from LP glomeruli (Figure 8).

### Expression Profile of the miR-200 Family and of miR-192 in Isolated Glomeruli

**Figure 9:**
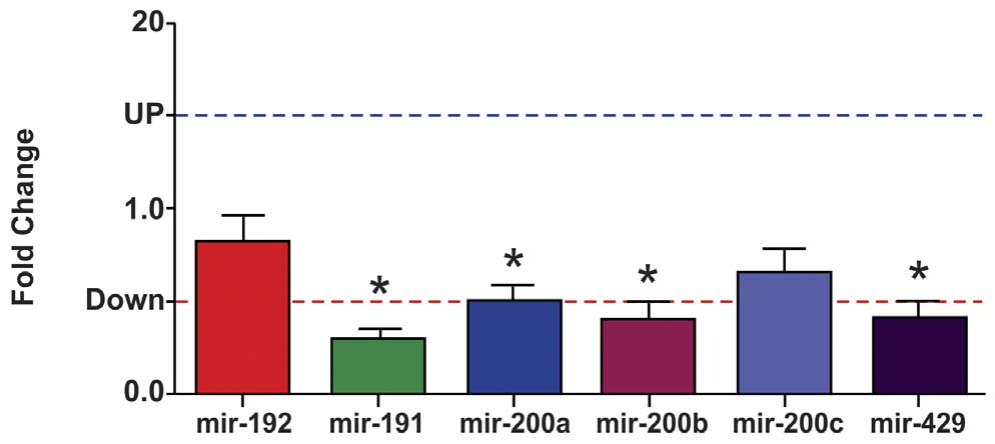
Expression of the miR-200 family and miR-192. **Expression levels of mir-192, mir-141, mir-200a, mir-200b, mir-200c and mir-429 estimated by TaqMan RT-qPCR in isolated glomeruli of 16-wk old LP rats.** The expression of each microRNA was normalized for U6 and U87 genes. Data are expressed as fold change (mean ± SD, n = 5) relative to control group (n = 5). *Significantly different from control group (P≤0.05).

The miR-200 family is divided into two groups according to their sequence seed: group 1 (miR-141 and miR-200a) and group 2 (miR-200b, miR-200c and miR-429) (38). In isolated glomeruli, of 16-wk-old LP animals, miR-141, miR-200a, miR-200b and miR-429 were significantly down-regulated compared to NP offspring (Figure 9).

### Gene Expression in Isolated Glomeruli

**Figure 10:**
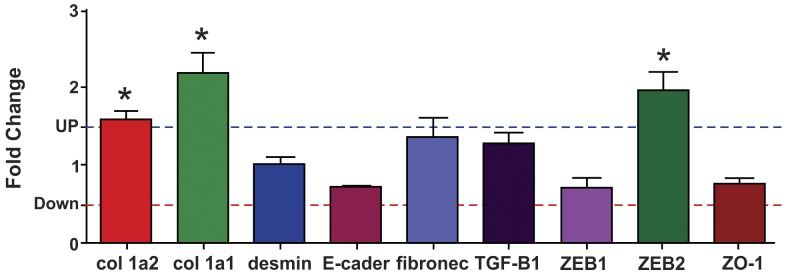
Expression levels of mRNAs. Expression levels of col 1α1, col 1α2, desmin, E-cadherin, fibronectin, TGF-β1, ZEB1, ZEB2 and ZO-1 estimated by SyBR green RT-qPCR in glomeruli of LP rats. The expression of each mRNA was normalized for GAPDH, β-actin and TBP genes. Data are expressed as fold change (mean ± SD, n = 5) relative to control group (n = 5). *Significantly different from control group (P≤0.05).

The miR-200 family members target both ZEB1 and ZEB2 (39). In LP offspring, the gene expression for collagen 1α1, collagen 1α2 and ZEB2 gene expression were significantly increased in isolated glomeruli (Figure 10). Surprisingly, unchanged findings for mRNA expression for desmin, E-cadherin, fibronectin, TGF-β1, ZEB1 and ZO-1 were found for both groups (Figure 10).

## Discussion

Brenner *et al*. (1998) proposed that congenital or programmed reduction in nephron number explains why some individuals are susceptible to hypertension and renal injury whereas others with sodium excess or diabetes seem relatively resistant under similar circumstances [24]. A reduction in nephron number and, therefore, in the whole-kidney glomerular filtration area would result in reduced sodium excretory capacity, enhanced susceptibility to hypertension and reduced renal reserve; thereby, the limiting compensation for renal injury may, at least in part, explain the higher prevalence of hypertension and renal disease observed in populations with high prevalence of low birth weight [2], [3], [25]–[27]. In this case, a reduced remaining glomerular number associated with increased glomerular volume is consistent with enhanced blood flow and hyperfiltration per unit of nephron. The long-time hyperfiltration process manifests, itself, as microalbuminuria and accelerated loss of renal function; findings that are highly prevalent among adult animals that had been of low birth weight (LBW) [2], [8].

In the current study, confirming the previous report, the birth weight of male LP offspring was 19.9% lower and, as previously reported, this is associated with 27% reduced nephron number and enlargement of remaining glomeruli [2], [8]. Our group has previously showed that, even when associated with decreased nephron number units, maternal food-restricted offspring keep a whole kidney with normal glomerular filtration rate (GFR) estimated by creatinine clearance, suggesting, in fact, a compensatory glomerular hyperfiltration despite a loss of efficiency on the filter barrier [2], [8]. The present study, taking into account the renal results and increased blood pressure in maternal low protein diet-intake model, corroborates to confirm Brenner’s hypothesis, by which, the hyperfiltration in LBW leads to glomerular hypertension and, in the future, to sustained renal function disorder [28].

Podocytes are highly terminally differentiated cells incapable of regenerative postnatal replication, with major and foot process interlinked by ultrathin slit diaphragms. Therefore, the loss of podocytes may lead to GBM “bare” areas, which represent potential starting points for irreversible glomerular injury [29], [30]. In LP offspring at 26-wk of age, it was observed the effacement of pedicels, the slit diaphragm was absent and there was an increase in GBM thickness [30]. The current study confirms these findings in 16-wk-old LP offspring, and shows a striking podocyte structural alteration in parallel with proteinuria and enhanced glomerular desmin expression, denoting a decreased efficiency on the filtration barrier in LP rats when compared with age-matched control offspring. Podocyte injury underlies most forms of proteinuric kidney diseases [31] and is an essential feature of progressive glomerular diseases [32]. As in glomeruli injury, the podocyte proliferation did not occur; we may hypothesize that PECs in the process of differentiating into podocytes, observed in the present study in 16-wk-old LP offspring, may be a re-supply of new cells for an injured nephron.

A new and interesting result is that transitional cells at the PEC/Podocyte interface have enhanced ZEB 2 expression. Taking into account the present study, we suppose that PEC type II epithelial-to-mesenchymal transition, induced by TGF-β1 and ZEB 2 overexpression, could be related to the loss of their well-defined cell-cell and cell-basement membrane contacts, thus allowing their migration to glomerular vascular stalk becoming morphologically similar to podocytes. A recent study have demonstrated that EMT plays a major role in imparting plasticity to terminally differentiated PECs by producing metastable cells with traits of kidney progenitors [33]. Also, Zhang *et al.* (2012) found in aging nephropathy an increased number of PECs and PECs expressing podocyte proteins [34].

By immunohistochemistry, the present study verified a striking enhanced glomerular expression of TGF-β1, fibronectin and type I collagen, intrinsically related to the fibrotic process. The current study shows that nearly 90% of LP animals have increased expression of these proteins in the renal cortex. Otherwise, we have hypothesized that hypertension development beyond 12-wk of age [2], [8] following maternal protein restriction associated with overload blood flow in remaining nephrons may be a preponderant factor for the development of fibrous process and altered glomerular ultrastructure in rat offspring.

As miRNAs have been suggested as playing a key role in a variety of kidney diseases, we investigated the expression of the miR-200 family and miR-192 in isolated glomeruli from LP compared to NP offspring. The current study has shown that LP rats present a significant downregulation of miR-141 (71%), miR-200a (50%), miR-200b (60%) and miR-429 (59%). Thus, in the glomeruli, in parallel to the enhanced expression of TGF-β1, a known inducer of EMT in epithelial cells [35], [36], we found downregulation of miR-200 family members and upregulation of ZEB2 that are, respectively, essential to maintain the normal epithelial phenotype and the EMT-inducing transcriptional factor [18], [20], [36], [37]. Additionally, in the present study, we demonstrated a significant enhancement in the expression of mesenchymal protein markers, including fibronectin, collagen 1_α_1 and collagen 1_α_2. Corroborating the present findings, Xiong *et al*. (2012) also verified downregulation of the miR-200 family induced by TGF-β1 in kidney cell culture [38]. In this way, Liu (2010) speculates that the transition of podocytes after injury may play a critical role on podocyte dysfunction, which, in turn, ultimately leads to defective glomerular filtration, proteinuria and glomerulosclerosis [5].

Prior findings indicate that glomerular podocytes undergo phenotypic conversion, characterized by loss of podocyte-specific markers and gain of transitional features, a process reminiscent of EMT [39]. In this study, we observed a great enhancement of the mRNA expression of collagen 1_α_1 (219%) and collagen 1_α_2 (157%), which was surprisingly accompanied by unchanged glomerular expression of desmin, E-cadherin and ZO-1 mRNA in LP offspring. Using culture of immortalized mouse podocyte, Li *et al*. (2008) showed that after TGF-β stimulation there was also loss of epithelial markers, such as ZO-1, and acquisition of mesenchymal markers, such as desmin, collagen I and fibronectin [39]. In these cases, we may not exclude the possibility of a post-transcriptional phenomenon in the gene pathway to reduce the desmin, E-cadherin and ZO-1 protein expression. Also, fibronectin and TGF-β1 mRNA expressions are unaltered, but, on the other hand, by immunohistochemistry, it was observed intensive immunolocalization for those proteins in glomeruli of LP rats. The production of interstitial matrix compounds suggests that podocytes have adapted a mesenchymal phenotype after injury, with profound change in their functions [39]. These authors address to the fact that podocytes may not undergo EMT in a synchronized fashion after injury and, therefore, would reflect different stages of podocyte EMT, and could be envisioned *in vivo*. Instead, as show in the current study as well in the previous one, approximately 80% of glomeruli of LP offspring showed enhanced expression of matrix compounds. TGF-β triggers tubular EMT and its expression is up-regulated in virtually every type of chronic kidney disease [40], [41] including in the LP programming model. We may affirm that EMT may be an early and predominant response of podocytes in most pathophysiological conditions. TGF-β1 protein expression, in whole kidney, is enhanced in the similar pattern that is observed for collagen type I, but this increase in protein was not followed by mRNA expression in glomeruli of LP offspring. TGF-β induces reduction in ZEB 1 expression. ZEB 1 is a repressor of col I e II genes [11], [12], [42] and, here, we did not find alteration in ZEB1 mRNA expression. Thus, we can suppose that ZEB2 has a major function in the EMT of glomerular cells.

Our contrasting findings highlight the complex nature of miRNA research. Kato *et al*. (2009) have considered that the effects of renal miRs may be cell type-specific, and that the miR signaling networks that mediate the effects of TGF β on different EMT types may not be the same [15]. However, so far, our study supports the recent review from Carew *et al*. (2012) stating that the precise role of miRNAs in regulating EMT and the pathogenesis of fibrosis remains a fertile area for research [13].

**Figure 11:**
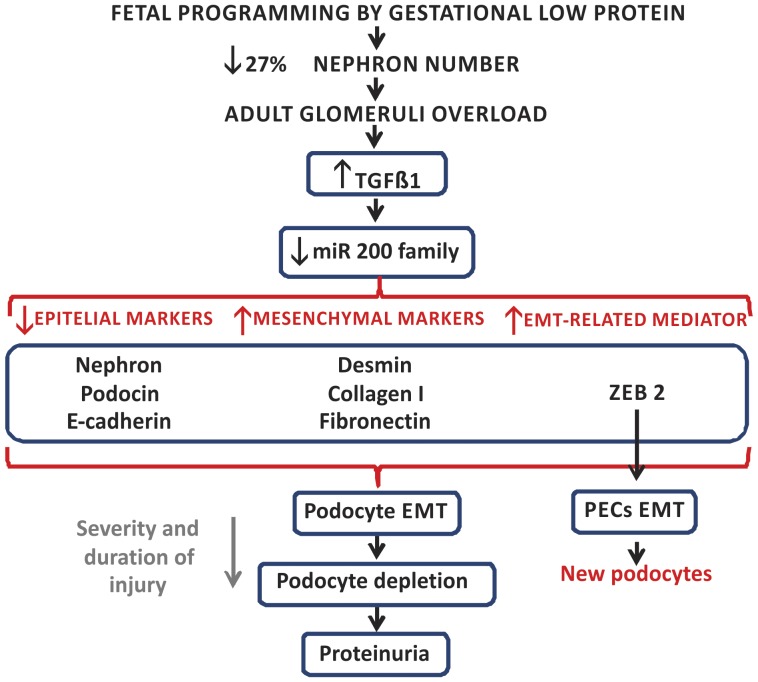
Schematic representation of proposed fetal programming consequences in cells of the renal corpuscles from adult rats. Gestational low protein diet leads to a reduction of 27% in nephron number and adult hypertension. We suppose that glomeruli overload is an initial insult that starts a cascade of events including an early inflammatory phase followed by a fibrogenic response. The downregulation of the microRNA-200 family, in response to enhanced TGFβ 1 expression, results in the reduction of epithelial markers and enhancement in mesenchymal markers and in ZEB 2, an EMT mediator. Podocytes will undergo EMT to escape apoptosis, which results in podocyte simplification, which leads to an impaired glomerular filtration barrier ensuring the onset of proteinuria. Depending of the severity and duration of injury, there may be a podocyte loss exacerbating proteinuria. PECs will also undergo EMT for differentiation into new podocytes in an attempt to attenuate the glomerular function loss (modified from Liu, 2010) [5].

Considering that the glomeruli are the first nephron structures affected by overflow and pressure overload, we may state that hypertension development following maternal protein restriction, associated with overload in remaining nephrons, could be a preponderant factor for the development of glomerular alterations such as those observed here (Figure 11). From our present knowledge, these are the first data showing renal miRNA modulation in the protein restriction model of fetal programming. The fetal-programmed adult rats showed pronounced structural glomerular disorders with an accentuated and advanced stage of fibrosis, which led us to state that the glomerular miR-200 family would be downregulated by TGF-β1 action inducing ZEB 2 expression that may subsequently cause glomerular EMT.

## Materials and Methods

### Animals and Diets

The experiments were approved by the ethical committee for experimental research at the Institute of Biosciences (292-CEEA) at the São Paulo State University. The general guidelines established by the Brazilian College of Animal Experimentation (COBEA) were followed throughout the investigation. The assays were conducted on age-matched, female offspring of sibling-mated Wistar Hannover rats (0.250–0.300 kg) with free access to water and standard rodent diet (Nuvital, Curitiba, PR, Brazil). Our local colonies originated from a breeding stock supplied by CEMIB/Unicamp, Campinas, SP, Brazil. Immediately after weaning at 3 weeks of age, animals were maintained under controlled temperature (25°C) and lighting conditions (0700 h-1900 h), with free access to tap water and standard rodent laboratory diet, and followed up to 12 weeks of age. The day that sperm were seen in the vaginal smear was designated as day 1 of pregnancy. Forty dams were maintained on isocaloric standard rodent laboratory with normal protein content [NP, n = 20], (17% protein) or low protein content [LP, n = 20] (6% protein) diet *ad libitum* intake throughout the entire pregnancy. All groups returned to the NP diet intake after delivery. Birth weight was measured. The male pups were followed and maintained with normal diet until 16 weeks old. Offspring from the NP (rats from different mothers) and LP (rats from different mothers) groups were used for morphological and molecular analysis. Groups were weighed and euthanized on the 16^th^ week of age. The kidneys were weighed and tissues samples were collected for MET, MEV, immunohistochemistry and western blot. Glomeruli were sieved from renal tissue as previously described [43] for real-time PCR analyzes.

### Measurement of Proteinuria

Sixteen-week-old male rats from the NP (n = 10) and LP (n = 10) groups received 15 mL of tap water load by gavage and, twenty minutes after it, were housed individually in metabolic cages, and spontaneously voided urine was collected over a 2-h period and immediately stored at −20°C until processing. The proteinuria was detected using the *Sensiprot Kit* (Labtest).

### SEM Studies

Sixteen-week-old male rats from the NP (n = 5) and LP (n = 5) groups were used. The rats were anesthetized with a mixture of ketamine (75 mg.kg-1body weight, i.p.) and xylazine (10 mg.kg-1body weight, i.p.) and perfused by the left carotid artery with saline containing heparin (5%) for 15 minutes under constant pressure. This was followed by perfusion with 0.1 M phosphate buffer (PB; pH 7.4) containing 4% (w/v) paraformaldehyde and 0.1 M sucrose for 25 min. After perfusion, renal cortical slices were immersed in Karnovsky’s solution (2% glutaraldehyde, 2% paraformaldehyde in 0.1 M phosphate buffer, pH 7.4) at 4°C overnight. After rinsing in phosphate buffer for 1 hour, the specimens were postfixed in buffered 1% OsO4 at 4°C in the dark for 2 hours and then immersed in a 2.3 M sucrose solution at 4°C overnight. The specimens were subsequently immersed for 30 min in liquid nitrogen and then fractured, washed in the same buffer, dehydrated in a graded acetone series, and critical-point dried. After identifying the fractured surface, specimens were mounted on stubs, sputtered with gold for 120 s, and examined and photographed with a scanning electron microscope operated at 10 kV.

### TEM Studies

After perfusion, the kidneys from the 16-wk old NP (n = 5) and LP (n = 5) rats were removed, and cortical slices were cut into small pieces, which were immersed in the same fixative with 0.1% tannic acid and 5% sucrose for 3 hours at room temperature. After rinsing in a sugar-saline solution (0.15 M NaCl, 0.2 M sucrose), the specimens were postfixed with 1% OsO4 at 4°C in the glucose-saline solution in the dark for 2 hours and then rinsed again in the glucose-saline solution. The samples were dehydrated in a graded ethanol series and embedded in Epon 812 resin at 60°C for 48 h. Thin sections (60–70 nm) were double-stained with uranyl acetate and lead citrate and were observed and photographed with a transmission electron microscope operated at 60 kV.

### Immunohistochemistry

After perfusion, the kidneys from the 16-wk old NP (n = 5) and LP (n = 5) rats were removed and placed in the same fixative for 2 h, followed by 70% alcohol until processed for paraffin inclusion or frozen in liquid nitrogen. The frozen and paraffin blocks were cut into 5-µm-thick sections. Paraffin sections were, overnight at 4°C, incubated with primary antibodies for anti-collagen I (Sigma), anti-fibronectin (Novo Castra), anti-TGF-β1, anti-E-cadherin, anti-nephrin, anti-desmin and anti-ZEB 2 (Santa Cruz). Secondary antibodies were used according to the primary antibody. Finally, sections were revealed with DAB, counterstained with Mayer’s hematoxylin, dehydrated and mounted. For immunofluorescence, the sections were incubated sequentially with: (1) phosphate-buffered saline (PBS) containing 5% milk for 45 min to minimize non-specific reactions, (2) anti-nephrin, at 4°C overnight, and (3) goat anti-mouse Alexa 488 antibody (Alexa) for 2 h at room temperature. After incubation, the sections were rinsed in 0.1 M PBS and cover-slipped with Vectashield anti-fading medium containing DAPI (Vector). No immunoreactivity was seen in control experiments in which one of the primary antibodies was omitted.

### Immunoblotting

Whole kidneys were obtained from NP (n = 5) and LP (n = 5) rats. The tissue was minced coarsely and homogenized immediately in 10 volumes of solubilization buffer (10 ml/L Triton-X 100, 100 mM Tris[hydroxymethyl]aminomethane (Tris) pH 7.4, 10 mM sodium pyrophosphate, 100 mM sodium fluoride, 10 mM ethylenediaminetetraacetic acid (EDTA), 10 mM sodium vanadate, 2 mM phenylmethylsulfonyl fluoride (PSMF) and 0.1 mg/ml aprotinin at 4°C), using a polytron PTA 20S generator (model PT 10/35, Brinkmann Instruments, Westbury, N.Y., USA) operated at maximum speed for 20 s. The tissue extracts were centrifuged at 11,000 rpm at 4°C for 40 min, and the supernatants were used as sample.

Protein quantification was performed using the Bradford method. For quantification, both tissue and total extract samples (250 µg of protein) were subjected to SDS-PAGE. After electrophoretic separation, proteins were transferred to nitrocellulose membranes and then blotted with specific antibody. The samples were treated with Laemmli buffer containing 100 mM dithiothreitol (DTT), heated in a boiling water bath for 4 min and subjected to 8% sodium dodecyl sulfate-polyacrylamide gel electrophoresis (SDS-PAGE) in a Bio-Rad minigel apparatus (Mini-Protean, Bio-Rad). Electrotransfer of proteins from the gel to the nitrocellulose membranes was performed for 90 min at 120 V (constant) in a Bio-Rad miniature transfer apparatus (Mini-Protean). The non-specific protein binding to the nitrocellulose was reduced by pre-incubating the filter for 2 h at 22°C in blocking buffer (5% non-fat dry milk, 10 mM Tris, 150 mM NaCl, and 0.02% Tween 20). Nitrocellulose blots were then incubated at 4°C overnight with primary antibodies diluted in blocking buffer (3% non-fat dry milk, 10 mM Tris, 150 mM NaCl, and 0.02% Tween 20) as follow: anti-nephrin or anti-podocin (Abcam), (1∶700). Immunoreactivity bands were detected using the enhanced chemiluminescence method (RPN 2108 ECL Western blotting analysis system; Amersham Biosciences). Images of the developed radiographs were scanned (Epson Stylus 3500) and band intensities were quantified by optical densitometry (Scion Image Corporation).

### Total RNA Extraction

RNA was extracted from isolated glomeruli pool from NP (n = 5) and LP (n = 5) rats using Trizol reagent (Invitrogen), according to the instructions specified by manufacturer. Total RNA quantity was determined by the absorbance at 260 nm using nanoVue spectrophotometer (GE healthcare, USA), and the RNA purity was assessed by the A 260 nm/A 280 nm and A 260 nm/A 230 nm ratios (acceptable when both ratios were >1.8). RNA Integrity was ensured by obtaining a RNA Integrity Number - RIN >8 with Agilent 2100 Bioanalyzer (Agilent Technologies, Germany).

### Reverse Transcription of miRNA and mRNA

cDNA was synthesized using TaqMan® microRNA Reverse Transcription kit (Life Technologies, USA), combined with Stem-loop RT Primers (Life Technologies, USA) and High Capacity RNA-to-cDNA Master Mix (Life Technologies, USA) according the manufacturer’s guidelines. For miRNA, 3 µl (10 ng) total RNA was mixed with specific primers (3 µl), dNTPs (100 mM), MultiScribe™ Reverse Transcriptase (50 µl), 10×RT Buffer, RNase inhibitor (20 µl) and completed up to 4.5 µl with H_2_O. The cycling conditions were: 16°C for 2 min, 42°C for 1 min, 50°C for 1 second and 85°C for 5 min. For mRNA, 10 µl total RNA was mixed with 4 µl Master Mix, 2 µl specific primers and completed up to 20 µl with H_2_O. The cycling conditions were: 25°C for 5 min, 42°C for 30 min and 85°C for 5 min.

### Real-time Quantitative PCR (miRNAs)

Each cDNA of miRNA-200 family (miR-200a, miR-200b, miR-200c, miR-141 and miR-429) and miR-192 was quantified by real-time quantitative PCR using ABI Prism 7900 Sequence Detection System (Life Technologies, USA). We used, for each reaction, 10 µl TaqMan® Universal PCR Master Mix, 2 µl TaqMan MicroRNA Assay Mix (Life Technologies, USA), 1.5 µl cDNA and completed up to 20 µl reaction volume. The cycling conditions were: 95°C for 10 minutes; 45 cycles of 95°C for 15 seconds and 60°C for 1 minute.

### Real-time Quantitative PCR (mRNAs)

For the analysis of expression level of ZEB1, ZEB2, desmin, fibronectin, ZO-1, E-cadherin, TGF-β1, col 1α1 and col 1α2, in isolated glomeruli, RT-qPCR was carried out with SYBR green Master Mix, using specific primers for each gene (Table 1). Reactions were set up in a total volume of 20 µL using 5 µl of cDNA (diluted 1∶100), 10 µL SYBR green Master Mix (Life Technologies, USA) and 2.5 µL of each specific primer (5 nM) and performed in the ABI Prism 7300 real-time PCR system (Life Technologies, USA). The cycling conditions were: 95°C for 10 minutes; 45 cycles of 95°C for 15 seconds and 60°C for 1 minute.

**Table 1:** Gene sequence studied in isolated glomeruli.

GENES	SEQUENCE (Forward)	SEQUENCE (Reverse)
**ZEB 1**	5′-CATTTGATTGAGCACATGCG-3′	5′-AGCGGTGATTCATGTGTTGAG-3′
**ZEB 2**	5′-CCCTTCTGCGACATAAATACGA-3′	5′-TGTGATTCATGTGCTGCGAGT-3′
**DESMIN**	5′-GCGTGACAACCTGATAGACG-3′	5′-GTTGGATTTCCTCCTGTAGTTTG-3′
**FIBRONECTIN**	5′-AGACCCCAGGCACCTATCAC-3′	5′-TGGCCGTTTCAGGAAGGTTG-3′
**TGFβ-1**	5′-GGACTCTCCACCTGCAAGAC-3′	5′-GACTGGCGAGCCTTAGTTTG-3′
**COL1α1**	5′-ACCTGTGTGTTCCCCACTCA-3′	5′-CTTCTCCTTGGGGTTTGGGC-3′
**COL1α2**	5′- ACAAGGTGCTCGTGGTTTCC -3′	5′- GCACCAGGCTGTCCTTTCAA -3′
**E-CADHERIN**	5′-ATGAGGTCGGTGCCCGTATT-3′	5′-CTCGTTGGTCTTGGGGTCTGT-3′
**ZO-1**	5′-GAGGCTTCAGAACGAGGCTATTT-3′	5′-CATGTCGGAGAGTAGAGGTTCGA-3′

Statistical Analyzes - Data obtained from this study are expressed as mean ± SEM. Statistical analyzes were performed using Student *t*-test from GraphPad Prism 5.01 (GraphPad Software, Inc., USA). Significance was set at p<0.05.

### Analysis of the Gene Expression

To analyze the differential expressions, the miRNA or mRNA levels obtained for each gene (Table 1) were compared with LP group with respect to the appropriated NP group. Normalization of miRNA expression was made using the expression of the snRNA U6 and snRNA U87 reference genes (Accession: NR_004394 and AF272707, respectively), and, for mRNA expression, the genes GAPDH, β-actin and TBP. Relative gene expression was evaluated using the comparative quantification method [43]. All relative quantifications were assessed using DataAssist software v 3.0, using the ΔΔCT method. PCR efficiencies calculated by linear regression from fluorescence increase in the exponential phase in the program LinRegPCR v 11.1 [44].
